# A *Drosophila* Insulin-like Peptide Promotes Growth during Nonfeeding States

**DOI:** 10.1016/j.devcel.2009.10.009

**Published:** 2009-12-15

**Authors:** Maija Slaidina, Rénald Delanoue, Sebastian Gronke, Linda Partridge, Pierre Léopold

**Affiliations:** 1 Institute for Developmental Biology and Cancer, University of Nice-Sophia Antipolis/Centre National de la Recherche Scientifique, Parc Valrose, 06108 Nice, France; 2 University College of London, Institute of Healthy Aging, GEE (Genetics, Evolution and Environment), University College London, Gower Street, London WC1E 6BT, UK

**Keywords:** DEVBIO

## Abstract

In metazoans, tissue growth relies on the availability of nutrients—stored internally or obtained from the environment—and the resulting activation of insulin/IGF signaling (IIS). In *Drosophila,* growth is mediated by seven *Drosophila* insulin-like peptides (Dilps), acting through a canonical IIS pathway. During the larval period, animals feed and Dilps produced by the brain couple nutrient uptake with systemic growth. We show here that, during metamorphosis, when feeding stops, a specific DILP (Dilp6) is produced by the fat body and relays the growth signal. Expression of *DILP6* during pupal development is controlled by the steroid hormone ecdysone. Remarkably, *DILP6* expression is also induced upon starvation, and both its developmental and environmental expression require the *Drosophila* FoxO transcription factor. This study reveals a specific class of ILPs induced upon metabolic stress that promotes growth in conditions of nutritional deprivation or following developmentally induced cessation of feeding.

## Introduction

Growth relies on the ability of cells and organisms to access nutrients. Nutrients can be obtained from diverse sources, such as from the environment through feeding, or from internal stores as with early embryos that develop from large eggs (O'Farrell, 2004). Accordingly, because alternate sources of nutrients are used during specific periods of development, organisms must be able to adapt their metabolic and growth programs to changes in the developmental or environmental energy context.

In complex animal species, growth is controlled by intermingled paracrine and endocrine regulatory processes, with organ and tissue growth governed by specific genetic programs that determine the target size and relative proportions of the species (Day and Lawrence, 2000). The output of these genetic programs is further modified by environmental cues, including nutrition. Variations in nutritional input can influence growth and metabolism via insulin/IGF signaling (IIS) (Underwood et al., 1994). In particular, when nutrients are abundant, IIS is maximally active and growth is limited solely by the organ-intrinsic program; upon nutrient shortage, in contrast, IIS becomes limiting and restricts the growth and metabolic parameters accordingly.

In mammals, the IIS system is split into two complementary and interacting subsystems that govern growth, metabolism, reproduction, and longevity (Nakae et al., 2001; Saltiel and Kahn, 2001). The first of these corresponds to circulating insulin levels, which control carbohydrate and fat metabolism, and the second is the GH/IGF-I axis, which regulates cell and tissue growth. Starvation lowers circulating IGF-I, in part through decreased transcription of the *IGF-I* gene in the liver (Thissen et al., 1994); this suggests that one major way in which starvation can affect growth is by reducing levels of circulating growth factors.

The function of IIS in growth control is remarkably conserved in insects, and in particular in *Drosophila*, where seven *Drosophila* insulin-like peptides (DILPs) have been identified (Géminard et al., 2006). The various *DILP* genes are expressed in different larval and adult tissues, suggesting that they carry nonredundant functions (Brogiolo et al., 2001). In particular, *DILP1*, *-2*, *-3*, and *-5* are expressed in specialized neurosecretory cells located in each brain hemisphere, called the insulin-producing cells (IPCs). Genetic ablation of these cells leads to severe larval growth deficits, hypertrehalosemia, and increased lifespan (Ikeya et al., 2002; Rulifson et al., 2002; Broughton et al., 2005).

One major role for IIS in insects is to couple growth with the animal's energy status. Indeed, total nutrient deprivation downregulates *DILP3* and *DILP5* transcription in the IPCs, although *DILP2* expression remains unchanged (Ikeya et al., 2002). Recent results indicate that variations in nutritional information are relayed by a nutrient sensor operating in the fat body, a larval organ that shares metabolic functions with the vertebrate white fat and liver. In particular, it has been shown that amino acid restriction triggers fat body-specific inhibition of the TOR complex1 (TORC1) (Colombani et al., 2003), a major cell-based nutrient-sensing pathway (Dann and Thomas, 2006; Wullschleger et al., 2006; Guertin and Sabatini, 2007). Inhibition of TORC1 in the fat body systemically reduces larval growth in part by blocking Dilp secretion from the brain IPCs (Géminard et al., 2009). Therefore, in line with the decreased levels of circulating IGF-I in vertebrates, starvation affects *Drosophila* growth by severely reducing brain-specific DILP function.

Interestingly, previous work has shown that protein starvation causes the growth arrest of endoreplicative larval tissues (ERTs), while only slowing the growth and proliferation of cells in the larval brain and in imaginal discs (Britton and Edgar, 1998). Similarly, generally reduced TOR signaling in the larva, which in many respects mimics the starvation state, strongly inhibits ERT growth, while generally sparing the imaginal tissues (ITs) that form the adult structures (Oldham et al., 2000). This suggests a protection mechanism whereby, under adverse nutrition conditions, the fat body allows larval resources to be reallocated to high-priority tissues like the imaginal discs. Significantly, such a mechanism would require that some ILPs are produced during starvation and activate IIS in the tissues that continue to grow.

Feeding arrest is also a programmed event during development. At the end of the larval period, animals undergo a stereotyped behavior called the wandering stage, when they migrate away from the food and prepare for pupariation. This developmentally induced starvation precedes the long pupal feeding arrest. During pupal development, larval tissues undergo intense remodeling. This process involves a major reallocation of resources, as future adult tissues form from ITs in a process that uses either nutrient stores that had accumulated in fat cells during larval life, or energy obtained from the degradation of obsolete larval tissues. Since organisms do not feed during this stage, no global growth or weight gain is observed; nevertheless, because tissue remodeling involves cell growth and proliferation, growth-promoting pathways presumably come into play (Ninov et al., 2009). The paradox of pursuing a growth program in a nonfeeding organism that is subjected to catabolic regulation could be circumvented by the induction of growth-promoting hormones upon feeding arrest.

We present here the characterization of a particular DILP, DILP6, which promotes growth during nonfeeding stages. The *DILP6* gene is expressed in fat body cells and is strongly induced during the wandering larval and pupal periods, as well as upon starvation. Reduced *DILP6* function results in a growth deficit during pupal development and an increased sensitivity to starvation in young adults. The sudden increase of *DILP6* expression at the onset of pupal development requires an endocrine signal that is provided by the steroid hormone ecdysone. In parallel, starvation increases *DILP6* expression through dFoxO-mediated feedback regulation of IIS. Therefore, DILP6 constitutes an IGF-like peptide with a specialized role in promoting growth during developmentally or environmentally induced nonfeeding states.

## Results

### IIS Promotes Pupal Growth

**Figure 1:**
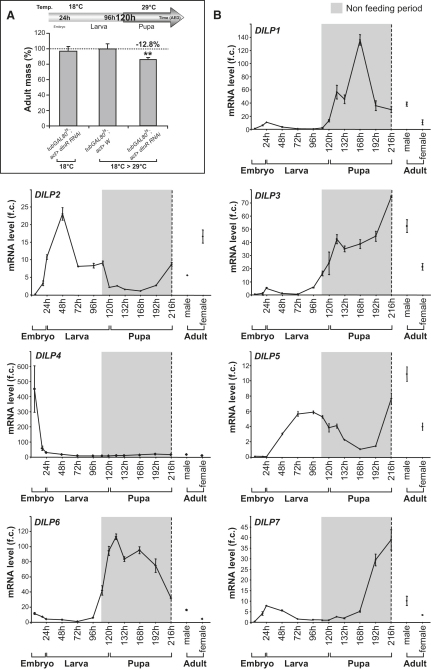
An Expression Time Course of *DILP* Genes during Development (A) At pupal stage, organismal growth requires insulin signaling. Temperature shift-up experiment (18°C–29°C) was carried out at the onset of the pupal stage (i.e., from 120 hr after egg deposition [AED] until adult eclosion) with *tubGal80^ts^*; *act > dInR RNAi*. *tubGal80^ts^*; *act > w* under the same temperature-shift program and *tubGal80^ts^*; *act > dInR RNAi* grown at restrictive temperature (18°C, normalized to *W*) were used as controls. Graph shows adult mass of animals in which *InR* was silenced during pupal stage compared to controls. Means ± SD are presented (n ≥ 50; ^∗∗^p < 0.01). (B) Expression of *DILP* genes during development. For each profile, fold changes are calculated relative to the minimal level. No cross-quantification is provided between the different DILP genes. Error bars represent SD.

Most studies concerning growth control in *Drosophila* have focused on the larval period. Within a time window of 4 days, the larval body weight increases 500-fold through the active conversion of nutrients into tissue mass. During this period, IIS plays a major role in coordinating nutrient intake and tissue growth: it promotes cell growth in postmitotic ERTs, cell growth coupled to proliferation in ITs, and the formation of substantial nutrient stores in fat cells. To investigate a possible role for IIS during the postfeeding developmental period, we used the *Gal4/Gal80^ts^* temporal conditional expression system (McGuire et al., 2004) to knock down InR specifically during pupal development. When transgenic animals containing the relevant constructs were raised at a restrictive temperature following the larval/pupal transition, the normal inhibition of Gal4 by Gal80 was relieved and the InR gene was silenced by RNAi. This targeted reduction of InR function during the pupal stage using this system resulted in smaller and lighter adults, with reduced appendages (Figure 1A; see Figure S1 available online). This demonstrates that IIS-dependent growth occurs during the nonfeeding developmental period in *Drosophila*.

### DILP6 Is a Fat Body-Derived Growth Inducer

To further investigate this phenomenon, we looked for specific *DILP* genes responsible for IIS activation in pupal tissues. Specifically, we used quantitative PCR (qRT-PCR) to examine the temporal expression patterns of the different *DILP* genes. This analysis revealed that, while the expression of most *DILP* genes decreases to basal levels at the end of the larval period, *DILP1*, *-3*, and *-6* are expressed at maximal levels during the pupal period (Figure 1B). We focused our attention on one of these genes in particular, *DILP6*, as its transcripts started accumulating at the wandering period, the late larval stage during which animals cease feeding and prepare for pupariation, and because it presented the highest rates of RNA accumulation during this period.

**Figure 2:**
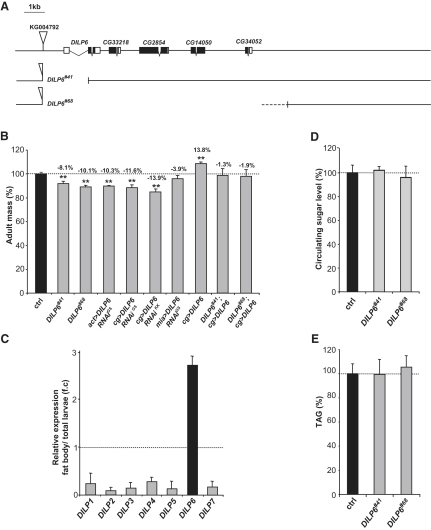
*DILP6* Is Required for Growth, but not for Carbohydrate and Lipid Metabolism (A) Schematic drawing of the genomic region around the *DILP6* gene. Two *DILP6* deletion alleles were generated by *P* element *KG004792* jump. Deletion allele *DILP6^#41^* removes the first exon and part of the first intron of the *dilp6* gene. Deletion allele *DILP6^#68^* removes *DILP6* and four adjacent nonannotated genes: CG33218, CG2854, CG14050, and CG34052. (B) *DILP6* loss of function were analyzed using either the two *DILP6* mutant alleles, *DILP6^#41^* and *DILP6^#68^*, at 25°C, or by silencing of *DILP6* ubiquitously (*act >*), in the fat body (*cg >*) and in the gut (*mia >*) at 29°C. *DILP6* overexpression in fat body was performed at 29°C. Graph represents means ± SD (n ≥ 50; ^∗∗^p < 0.01). (C) Relative expression of *DILP* genes assessed by qRT-PCR analysis in the fat body compared to total larva at wandering stage (110 hr AED). Graph represents means ± SD (n = 3). (D) Circulating carbohydrate levels in hemolymph of *DILP6* mutants compared to wild-type animals. Graph represents means ± SD (n ≥ 8). (E) TAG content of *DILP6* mutant compared to wild-type larvae. Graph represents means ± SD (n ≥ 6).

We first characterized two *DILP6* deletion mutants that were generated by imprecise excision of a *P* element inserted into the 5′ region of the gene. Deletion #41 removes part of the 5′ region as well as exon 1 of the *DILP6* gene, and deletion #68 removes the entire *DILP6* gene, plus four additional annotated transcription units downstream of *DILP6* (Figure 2A). No *DILP6* transcripts were detected in *DILP6^#68^* mutant larvae, whereas qRT-PCR detected sequences corresponding to second and third exons in *DILP6^#41^* larvae (Figure S2B). The two deletions produced indistinguishable phenotypes, with viable adults presenting a 8%–10% reduction in mass relative to sibling controls; this reduction could be fully rescued by targeted expression of the *DILP6* gene in the fat body (Figure 2B). The *DILP6^#41^* deletion was thus considered to be a strong hypomorph or null mutation for *DILP6*, and was used for our phenotypic analysis. Molecular analysis of *DILP6* transcripts by 5′-RACE for the *DILP6^#41^* mutant revealed the existence of two transcripts containing the starting AUG of the *DILP6* ORF with additional 5′ ORFs that could reduce the translation efficiency of *DILP6* and explain the observed phenotype (Figure S2A).

The growth defect observed with *DILP6* loss of function suggested that *DILP6* is not fully redundant with the other *DILP* genes, perhaps because of its specific function or timing of expression. Nevertheless, the levels of other *DILP* genes, especially *DILP1*, were elevated in *DILP6* mutant animals, suggesting that compensatory mechanisms may exist that may reduce the severity of *DILP6* loss of function (Figure S2B). Interestingly, the growth defect observed in *DILP6* mutants is not accompanied by a developmental delay (data not shown), indicating that the growth impairment occurs after the animals have passed the “critical period,” and can no longer compensate for growth deficits by extending the duration of larval development (Mirth and Riddiford, 2007).

*DILP6* is highly expressed in the fat body, a larval tissue that orchestrates the nutrient response and coordinates growth and metabolic functions (Figure 2C), and expressed at low levels in the gut and brain (Figure S2C). Specifically silencing *DILP6* in the fat body using the *cg-GAL4* (*cg>*) driver and two distinct *DILP6* RNAi constructs produced systemic growth defects that were similar to what was observed in the deletion mutant, indicating that the most important site of *DILP6* production is in the fat body. The silencing of *DILP6* in other larval tissues had no effects on systemic growth (Figure 2B and data not shown). Conversely, overexpressing *DILP6* in fat body cells increased systemic growth, indicating that *DILP6* is a bona fide growth inducer (Figure 2B). Surprisingly, the levels of circulating carbohydrates (trehalose and glucose) and triacylglycerides (TAGs) were not modified in *DILP6* mutant larvae at the wandering stage, suggesting that *DILP6* function, although limiting for growth control, is dispensable for metabolic regulation (Figures 2D and 2E).

### DILP6 Is Required for Growth during Nonfeeding Developmental Stages

**Figure 3:**
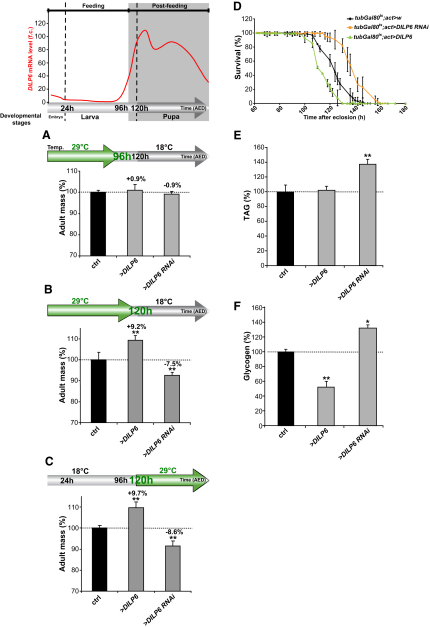
DILP6 Activates Growth from Postfeeding Larval Development until Adult Emergence (A–C) Changes in *DILP6* expression affect animal growth during late larval and pupal stages, but not during early larval development. *tub-Gal80^ts^*, *act > DILP6* or *tub-Gal80^ts^*, *act >, DILP6-RNAi* flies were kept at restrictive temperature (29°C) until 96 hr AED in order to silence or overexpress *DILP6* during early larval development. A shift-down to permissive temperature (18°C) at 96 hr AED has no effect on adult mass (A). When the temperature shift occurs at the larval-pupal transition (120 hr AED), changing *DILP6* expression induces significant effects on animal mass (B). Temperature shift-up from 120 hr AED until adult emergence affects animal mass (C). Adult mass is compared to *tub-Gal80^ts^*; *act > w* as control exposed to identical temperature-shift programs. Graph represents means ± SD (n ≥ 100; ^∗∗^p < 0.01). (D) Measurement of starvation resistance of newly emerged flies where *DILP6* was overexpressed or silenced from 120 hr AED until adult emergence. Starvation was performed at 18°C to inhibit Gal4 activity (error bars represent SD; n ≥ 84). (E and F) Measurement of TAG (E) and glycogen (F) contents of newly emerged flies where *DILP6* was overexpressed or silenced from 120 hr AED until adult emergence. Graph represents means ± SD (n ≥ 3); ^∗^p < 0.05, ^∗∗^p < 0.01.

We next explored the requirement for *DILP6* function during development using the *Gal4/Gal80^ts^* induction system. Specifically, *DILP6* function was temporally controlled using the ubiquitous *actin-Gal4* driver and the temperature-sensitive Gal4 inhibitor, *Gal80^ts^*, to express either a *UAS-DILP6 RNAi* or a *UAS-DILP6* construct at various times during development. We first observed that silencing *DILP6* before the wandering stage did not affect the final adult mass (Figure 3A). By contrast, extending *DILP6* silencing up to pupariation reduced the adult mass, indicating that *DILP6* function starts being required at the wandering stage (Figure 3B). This requirement extends into pupal development, as selective silencing of *DILP6* during the pupal stage also led to a growth defect (Figure 3C). Interestingly, increasing *DILP6* expression in wandering larvae and in pupae promoted growth, whereas it had no effect in early larvae (Figures 3A–3C). Together, these results indicate that *DILP6* is required during late larval and pupal development, and that its function is not limiting during earlier larval stages. These results were consistent with the normal timing of expression of *DILP6* and established that this ILP promotes growth specifically during nonfeeding periods of development.

In pupae, the supply of nutrients is limited to larval stores and cannot be renewed through feeding. The extent of growth during this period is determined both by the availability of nutrient stores and the level of circulating growth inducers. In order to assess the physiological significance of *DILP6*-mediated pupal growth control, we examined the effects of altering pupal growth by *DILP6* overexpression or silencing on the fitness of emerging adults. Large adults produced by increased *DILP6* expression in pupae presented reduced glycogen levels and were less resistant to starvation upon emergence. Conversely, small adults produced by lowered *DILP6* expression had increased glycogen and TAG levels and survived longer under starvation conditions (Figures 3D–3F). This indicates that, in the absence of nutrient uptake, DILP6 levels determine the balance between growth and resource storage, therefore indirectly influencing the metabolic state in the young adult.

### Ecdysone Controls the Developmental Expression of *DILP6*

**Figure 4:**
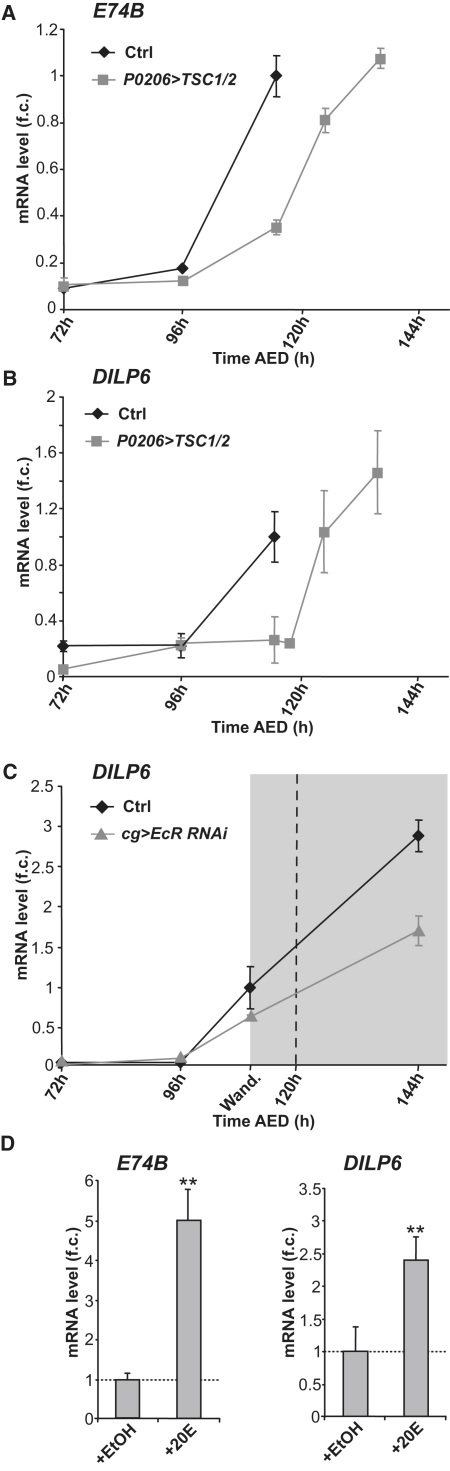
*DILP6* Expression Is Developmentally Controlled by Ecdysone (A) Overexpression of *TSC1/2* in the ring gland (*P0206 > TSC1/2*) delays the ecdysone peak: *E74B*, a direct target of ecdysone, is expressed with 24–30 hr delay compared with control larvae (*P0206 > w*) (see Layalle et al., 2008 for details). (B) Under the same experimental conditions, expression of *DILP6* is also delayed. mRNA levels are relative to control at 110 hr AED; error bars represent SD. (C) Measurement of *DILP6* expression by qRT-PCR in early (72 hr AED), mid (96 hr AED), and late (110 hr AED) third instar larva and in pupa. Control and *EcR* silencing in fat body cell (*cg > EcR RNAi*) conditions are shown. Fold changes are relative to control at 110 hr AED; error bars represent SD. (D) Measurement by qRT-PCR of *E74B* and *DILP6* expressions in dissected fat bodies incubated with 0.2% ethanol (+EtOH) or 2 μM 20E in 0.2% ethanol (+20E). Fold changes are relative to ethanol-treated control; error bars represent SD; ^∗^p < 0.05, ^∗∗^p < 0.01.

The rapid increase in *DILP6* expression during the late larval stage, as well as its sustained expression during the pupal stage, coincides with the increase in ecdysone titers at the larval/pupal transition. Therefore, we checked whether the developmental expression of *DILP6* could be controlled by ecdysone. For this, we reduced the level of TOR signaling in the prothoracic gland (the site of ecdysone production), a condition known to delay the peak of ecdysone production by 24 hr (Layalle et al., 2008). The effect on ecdysone production was confirmed by observing a 24 hr delay in the transcriptional induction of *E74B*, an early ecdysone-induced response gene (Figure 4A). Significantly, the profile of *DILP6* expression was delayed to the same extent, suggesting that the increase in *DILP6* levels that occurs at the end of larval development is controlled by the burst of ecdysone (Figure 4B). To further test our hypothesis, we specifically reduced the levels of ecdysone signaling in fat body cells by silencing the gene encoding the ecdysone receptor (EcR) (King-Jones and Thummel, 2005). EcR silencing in the fat body reduced *DILP6* transcript accumulation at the larval wandering stage and in pupae (Figure 4C). Finally, the addition of 20E to larval fat body explants cultured ex vivo was sufficient to induce *DILP6* transcription in fat cells (Figure 4D). Together, these results indicated that *DILP6* expression is driven by the ecdysone signal at the end of larval development, and that the main target tissue for this signal is the fat body.

### *DILP6* Is Induced upon Nutrition Shortage

**Figure 5:**
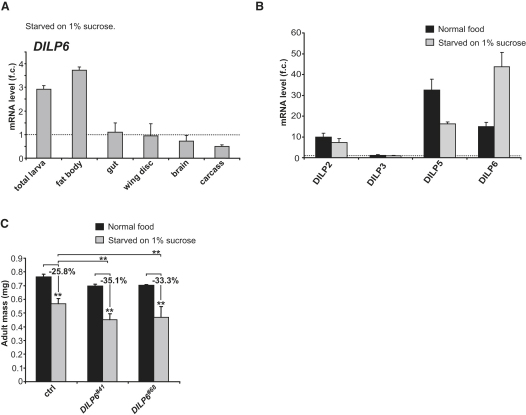
DILP6 Regulates Growth during Starvation (A) Measurement of *DILP6* expression by qRT-PCR in total larva or dissected organs from animals reared either in fed or starved conditions. Fold changes are relative to fed conditions; error bars represent SD. (B) Quantification of *DILP2*, *DILP3*, *DILP5*, and *DILP6* expression relative to *RP49* in fed and starved larvae. Fold changes are relative to *DILP3* expression in fed conditions. Larvae were starved at 72 hr AED for 16 hr on PBS 1% sucrose; error bars represent SD. (C) Measurement of adult mass of control animals and *DILP6* mutants (*DILP6^#41^* and *DILP6^#68^*) under fed and starved conditions. *DILP6* mutants display an aggravated loss of mass upon starvation compared with controls. Graph represents means ± SD (n ≥ 80; ^∗∗^p < 0.01).

*DILP6* expression is strongly induced in wandering larvae and in pupae, two developmental stages during which animals do not feed. This observation prompted us to ask whether *DILP6* expression could also be induced upon starvation. Indeed, when early feeding larvae were transferred overnight to agarose plates containing only 1% sucrose and no amino acid source, the global level of *DILP6* mRNA was strongly increased. Detailed analysis of dissected tissues revealed that *DILP6* expression was specifically increased in fat cells (Figure 5A). Moreover, the comparison of global expression levels of several *DILP* genes reveals that *DILP6* is expressed at higher levels than *DILP2*, *-3*, and *-5* under starvation (Figure 5B). To assess the physiological relevance of this regulation, we next examined the effects of starvation on organismal growth in a *DILP6* mutant background. Larvae were transferred to 1% sucrose/agarose medium at 96 hr after egg deposition (AED), and the weight of emerging adults was measured. While control adults raised under these conditions weighed 25% less than fed controls, *DILP6* mutants weighed 33% (*DILP6^#68^*) and 35% (*DILP6^#41^*) less than fed mutant animals (Figure 5C). This indicates that growth of *DILP6* mutants is compromised relative to control animals upon starvation, and therefore that the increase in *DILP6* expression observed upon starvation protects larvae from drastic impairments of organismal growth.

### dFoxO Controls Developmental and Environmental Expression of *DILP6*

**Figure 6:**
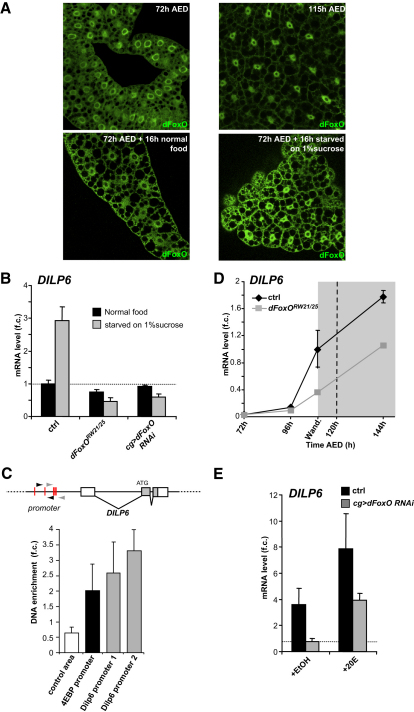
dFoxO Controls *DILP6* Expression upon Starvation (A) Immunostainings showing dFoxO protein (green) accumulating in the nuclei of fat body cells at 110 hr AED compared to 72 hr AED (top panels). At 72 hr AED, starvation also provokes an increase in dFoxO accumulation in fat body cell nuclei (bottom panels). (B) Measurement of *DILP6* expression by qRT-PCR in fed and starved conditions of wild-type, *dFoxO* mutant, and fat body-specific *dFoxO* knockdown larvae. Fold changes are relative to fed controls; error bars represent SD. (C) Chromatin immunoprecipitation (ChIP) performed on starved animals using either anti-dFoxO antibody or preimmune serum as a mock ChIP. Using two set of primers (gray and black arrowheads), qPCR shows that dFoxO binds to the *DILP6* promoter. Primers for the 4EBP promoter and for a nonrelated genomic area were used as positive and negative controls, respectively. (D) Developmental *DILP6* expression in *dFoxO* mutant animals. Although basal levels are reduced, *DILP6* expression is still induced at the larval/pupal transition. Fold changes are relative to control at 110 hr AED; error bars represent SD. (E) Measurement of *DILP6* expression by qRT-PCR in dissected fat bodies from control and *cg > dFoxO RNAi* animals incubated with 2 μM 20E (+20E) compared to controls treated with 0.2% ethanol (+EtOH). Fold changes are relative to ethanol-treated control; error bars represent SD.

*DILP6* transcription is specifically activated in nonfeeding conditions, and can be either developmentally or environmentally induced. Because larval fat body cells also accumulate the dFoxO transcription factor in its active, nuclear form both at the wandering stage and under starvation (Figure 6A), we investigated the possibility that both the environmental and developmental induction of *DILP6* expression involve dFoxO activity. We first examined the induction of *DILP6* expression by starvation in a *dFoxO* mutant background, and found that it was abolished (Figure 6B). These results were confirmed by knocking down *dFoxO* expression in the fat body, indicating that dFoxO function is autonomously required in fat cells for the induction of *DILP6* (Figure 6B). Analysis of the upstream region of the *DILP6* gene revealed the presence of several dFoxO binding sites, and chromatin immunoprecipitation experiments using dFoxO-specific antibodies yielded a genomic fragment of the *DILP6* gene containing these sites (Figure 6C). We conclude from these experiments that *DILP6* is a direct transcriptional target of dFoxO, and that dFoxO is required for increased *DILP6* expression upon starvation in fat body cells.

We then tested whether the developmental induction of *DILP6* also requires dFoxO function. For this, we observed the developmental profile of *DILP6* expression in larvae deprived of dFoxO. The rise in *DILP6* expression observed at the larval/pupal transition was delayed in *dFoxO*-null mutant larvae, but *DILP6* transcripts eventually reached intermediate levels, suggesting that, while dFoxO is required for the timely developmental expression of *DILP6*, other factors also contribute to its activation (Figure 6D). To specifically test the requirement for dFoxO in the induction of *DILP6* by 20E, we added 20E to fat bodies dissected from control or *cg > dFoxO RNAi* larvae, and measured *DILP6* levels by qRT-PCR. We observed that 20E was still able to induce *DILP6* expression in the absence of dFoxO, albeit at lower levels than in controls (Figure 6E). These ex vivo results confirmed our in vivo expression data, and indicate that dFoxO is required for normal *DILP6* expression induced either developmentally or by starvation. They also revealed that additional effectors other than dFoxO contribute to the induction of *DILP6* by 20E.

## Discussion

During the successive stages of development, organisms use alternate sources of nutrients to support tissue growth and morphogenesis. In *Drosophila*, embryonic tissues develop using maternal stores accumulated in the egg in the form of yolk. Larval development follows, with a major growth program relying on the animals' capacity to obtain nutrients from the environment. Finally, during the pupal stage, animals do not feed, and a large quantity of nutrients stored in fat cells allows pupae to prolong growth and finalize the development of adult structures. On top of these basic developmental strategies, feeding larvae have evolved additional buffering mechanisms to protect growing tissues from sudden variations in environmental energy supplies. Notably, brain ILPs promote larval growth and allow the coupling of growth to nutritional input. Their expression and secretion from brain IPCs decrease upon starvation (Géminard et al., 2009), and several brain DILPs show only residual expression in the pupa (see Figure 1). Therefore, there must be a distinct set of growth inducers that take the lead to activate growth in the pupa and upon nutritional stress. We show here that, in both of these contexts, a physiological switch takes place that triggers the activation of *DILP6*, a member of a distinct class of ILPs devoted to growth during nonfeeding periods.

The *DILP1* and *DILP3* genes are also expressed during pupal development, suggesting that they may act in concert with *DILP6*. Individual knockout of either of these two *DILP* genes only produces marginal growth defects (S.G. and L.P., unpublished data), suggesting that there is a high level of redundancy between them or with *DILP6*. Our observation that *DILP1* expression increases two-fold in *DILP6* mutant larvae suggests a possible compensatory mechanism that could partially suppress the growth impairment observed in *DILP6* mutants. The functional class of ILPs represented by DILP6 may be conserved in other insect species, as an ecdysone-induced, fat-body-specific ILP has recently been described in *Bombyx mori* (Okamoto et al., 2009).

The developmental and environmental induction of *DILP6* involves overlapping mechanisms. First, in response to nutrient deprivation, the IIS component, dFoxO, provokes a burst of *DILP6* transcription, thereby linking *DILP6* expression with the nutritional status of the animal. This represents a feedback regulation on IIS, as dFoxO, an inhibitor in the IIS pathway, induces the expression of *DILP6,* an activator of IIS. Interestingly, expression of *DILP3* in the adult was also recently shown to depend on *dFoxO* function (Broughton et al., 2008), suggesting that other *DILP* genes in this subclass are subjected to similar controls.

DILP6 does not appear to be effective as a paracrine/autocrine factor for fat cells. Indeed, fat cells of starved larvae, which express high levels of *DILP6*, undergo extensive autophagic transformation, even though autophagy has been shown to be blocked in these cells by IIS activation (Rusten et al., 2004; Scott et al., 2004). In addition, overexpression of *DILP6* in the fat body of starved larvae does not prevent autophagy (M.S. and P.L., not shown).

More generally, ERTs present stronger growth inhibition in response to starvation than do ITs. The role of a starvation-specific ILP that is induced upon nutritional stress could be to reroute energy stores toward high-priority organs and tissues, such as those responsible for the formation of the future adult. The specific action of DILP6 on imaginal cells could contribute to this diversified behavior, although this would require that ITs are more receptive to the DILP6 signal than are ERTs, at least upon starvation. Such differences in the response of ERTs and ITs to the DILP signal, combined with the production of specific DILPs upon starvation, could constitute a bona fide mechanism for the specific allocation of spare resources to ITs under nutritional stress. However, the mechanisms for such a biased response need to be elucidated.

At the end of larval development, animals stop feeding and prepare for pupal development. We show here that tissue remodeling in the pupa involves IIS-dependent growth, and that *DILP6* is specifically expressed and required for growth during this period. The transition from larval to pupal development is controlled by the steroid hormone ecdysone (20E), and we also show that 20E is required for proper *DILP6* induction at the larval/pupal transition. In view of the absence of obvious EcR/Usp binding sites in the 5′ region of the *DILP6* gene, as well as our previous demonstration that EcR signaling controls dFoxO nuclear localization (Colombani et al., 2005), we hypothesized that dFoxO could mediate the ecdysone-dependent expression of *DILP6*. However, both genetics and ex vivo experiments on dissected fat bodies indicate that, although dFoxO appears to contribute to the developmental induction of *DILP6* at the larval/pupal transition, it is not required for the 20E-induced expression of *DILP6*. In an accompanying manuscript, Okamoto et al. (2009) report that 20E-induced expression of *DILP6* is not affected by cycloheximide, suggesting that the transcriptional induction of *DILP6* by EcR/Usp is direct.

We have previously shown that ecdysone has a growth-inhibitory function during larval development (Colombani et al., 2005). Indeed, increased basal levels of circulating ecdysone in larvae can reduce the growth rate and, conversely, decreased basal ecdysone levels can increase the growth rate. Although the mechanisms underlying this relationship are not yet fully understood, we have established that the levels of ecdysone produced experimentally in these experiments remain close to basal levels, and are insufficient to modify *DILP6* expression (M.S., R.D., and P.L., unpublished data). Therefore, while basal levels of ecdysone can inhibit systemic growth through an unknown mechanism, high ecdysone levels at the larval/pupal transition can induce *DILP6*, and thus systemically activate IIS.

One puzzling observation reported here is that the modification of *DILP6* expression in pupae can alter the adult mass as well as the resistance of animals to starvation at eclosion. How can *DILP6* overexpression in pupae increase adult mass if the mass of the pupa is fixed at the end of larval life? One possible explanation is that DILP6 participates in a tradeoff between the construction of adult tissues and the maintenance of energy stores in the pupa. Indeed, the levels of both TAG and glycogen stores in the young adult are affected by DILP6 levels in the pupa. In this line, recent reports indicate that, under optimal conditions, not all nutrients are used by the pupa, and part of the energy is conserved to provide sustenance during the early period of adult life that precedes feeding. Some larval fat body cells are still present in early adults, and provide energy until feeding begins. Suppressing the death of these cells increases the energy stores and enhances the resistance of young adults to starvation (Aguila et al., 2007). *DILP6* knockdown in pupae has a similar effect: less energy is used by the pupa to build tissues, meaning that the adult ecloses with a smaller body, but with greater energy stores to help overcome early nutritional stress. *DILP6* overexpression has the opposite effect. Our results therefore indicate that DILP6 sets the energy balance in pupae by promoting tissue growth, while sparing an energy pool that can be used by the young adult.

DILP6 shares some specific features with vertebrate IGF-I that distinguish both of them from insulin. DILP6 peptide sequence does not present obvious cleavage sites for an internal C peptide (Brogiolo et al., 2001). It is produced in the fat body, a tissue sharing common functions with the vertebrate liver, where IGF-I is mainly produced. *DILP6* mutant animals present growth defects without obvious metabolic changes, suggesting that DILP6 might have an exclusive growth function. Finally, the induction of growth factor production under conditions of energy stress is also relevant to cancer biology. Indeed, IGF-I and IGF-II are frequently expressed within neoplastic tissue. It is suspected that they act as autocrine and paracrine growth factors within tumors, allowing tumor cells to evade nutritional shortage and acquire survival properties (Pollak, 2008). The induction of *DILP6* under starvation and its preferential targeting to ITs instead of ERTs could represent an interesting parallel to the induction of IGFs in tumor cells, where the selective action of growth factors can promote growth and survival of specific tissues in a nonfavorable environment.

## Experimental Procedures

### Fly Strains and Food

The following fly lines were used: *w^1118^* as a control; the fat body driver *cg-Gal4* (Takata et al., 2004), the gut driver *myo1D-GAL4* (DGRC, Kyoto); the ring gland driver *P0206-GAL4* and *UAS-EcR RNAi* (Colombani et al., 2005); *UAS-DILP6* (Ikeya et al., 2002); *UAS-TSC1* and *UAS-TSC2* (Tapon et al., 2001); *UAS-PI3K* and *UAS-PI3K^DN^* (Leevers et al., 1996); *UAS DILP6 RNAi^GS^* and *DILP6 RNAi^KK^* (VDRC, Vienna, Austria); and *dFoxo^Rw21^* and *dFoxo^Rw25^* (Junger et al., 2003). Other lines were obtained from the Bloomington Stock Center.

Animals were reared at 18°C, 25°C, or 29°C on 2× food containing, per liter: 34 g inactivated yeast powder, 83 g corn flour, 10 g agar, 60 g white sugar, 4.6 g Nipagin M (in ethanol). Starvation experiments were performed overnight or as indicated by transferring larvae on PBS 1% sucrose at 72 hr AED for gene expression measurements and EdU incorporation in brain, or 96 hr AED for EdU incorporation in wing discs. For adult mass measurements, animals were transferred to PBS 1% sucrose at 96 hr AED until adult eclosion.

The 0.1× yeast medium for the ChIP experiment was comprised of 1.7 g inactivated yeast powder, 83 g corn flour, 10 g agar, 60 g white sugar, and 4.6 g Nipagin M (in ethanol).

### Weighing Flies

First instar larvae were collected 24 hr AED (4 hr egg collections) and reared 40–50 animals per tube. Groups of 25 adult males were weighed with an Adventurer Pro Precision balance (Ohaus).

### qRT-PCR

Animal and tissue samples were dissected and/or collected in 1× PBS and frozen in liquid nitrogen. Total RNA of larvae, fat bodies, gut, and body walls was extracted using QIAGEN RNeasy lipid tissue Minikit according to the manufacturer's protocol. Total RNA of ITs and brain were extracted using QIAGEN RNeasy Microkit according to the manufacturer's protocol. RNA samples (1–2 μg/reaction) were reverse transcribed using SuperScript II reverse transcriptase (Invitrogen), and the generated cDNA used for real-time RT-PCR (StepOne Plus; Applied Biosystems) using PowerSYBRGreen PCR mastermix (Applied Biosystems) with 2.4 ng of cDNA template and a primer concentration of 300 nM. Samples were normalized with *RP49*. Two or three independent biological samples were collected for each experiment, and triplicate measurements were conducted. For each gene, two independent sets of primers were used. Primers were designed using the PrimerExpress software (Applied Biosystems), and their sequences are available on request.

### Circulating Sugar Quantification

Hemolymph from eight groups of 10 larvae 110 hr AED was used for each condition. Hemolymph was diluted (1:10) in homogenization buffer (137 mM NaCl, 2.7 mM KCl, 5 mM Tris [pH 6.6]), heated for 5 min at 70°C, and trehalose was converted into glucose after incubation with porcine trehalase (T8778; Sigma) at 37°C overnight. Total glucose was measured using the Thermo Glucose GOD-POD assay kit (Thermo Fisher Scientific). Quantifications were performed using a Sunrise spectrophotometer plate reader at 510 nm (Tecan).

### TAG Quantification

Three larvae 110 hr AED or four adult flies per experiment were frozen in liquid nitrogen. Lysis was performed using TissueLyser II (QIAGEN) in PBS 0.2% Tween 20 containing protease inhibitor cocktail (Roche). Lysates were cleared by centrifugation. An aliquot was kept for measurement of protein concentration; the rest of the lysate was heated for 5 min at 70°C. TAG was measured using the Term Triglycerides assay kit (Thermo Fisher Scientific). Quantifications were performed using a Sunrise spectrophotometer plate reader at 510 nm. Protein concentration was measured using the Thermo Total Protein assay kit (Thermo Fisher Scientific), and quantifications were performed using a Sunrise spectrophotometer plate reader at 540 nm. TAG level was normalized to protein level.

### Glycogen Quantification

Four adult flies were frozen in liquid nitrogen. Lysis was performed using TissueLyser II (QIAGEN) in PBS 0.2% Tween 20 containing protease inhibitor cocktail. Lysates were cleared by centrifugation. An aliquot was kept for measurement of protein concentration; the rest of the lysate was heated for 5 min at 70°C. Lysate was incubated with amyloglucosidase (Roche) in 50 mM sodium acetate buffer (pH 4.2) for 1 hr at 37°C to convert glycogen to glucose, then glucose level was measured using the Thermo Glucose GOD-POD assay kit. Quantifications and normalizations to protein level were performed as described in TAG Quantification.

### Adult Fly Starvation Resistance

Starvation resistance measurements were performed using a method based on that of (Aguila et al., 2007). Newborn flies were collected every hour and transferred into tubes containing 1.3% agarose in PBS. Dead flies were counted every 2–4 hr.

### Ex Vivo Fat Body Cultures

Animals 96 hr AED were dissected in Schneider medium (PAA, France) containing 8.5% FBS (Lonza, Belgium). Larvae were inverted and gut was removed. A total of 15–20 specimens per condition were transferred to 2 ml tubes with 2 μM 20E (Sigma) or 0.2% ethanol in Schneider medium containing 8.5% FBS and incubated for 5 hr at 29°C on a shaker. Fat bodies were dissected and frozen in liquid nitrogen.

### Immunofluorescence on Larval Tissues

Tissues were dissected from wandering larvae or as otherwise indicated in 1× PBS, fixed in 3.7% formaldehyde (Sigma) in PBS for 20 min at room temperature, and extensively washed in PBS containing 0.3% Triton X-100 (PBT). Tissues were then blocked for 1 hr in PBT containing 10% FCS. Primary antibodies were incubated overnight at 4°C. Secondary antibodies were incubated for 2 hr at room temperature. DNA was stained with Hoechst 33,258 (1 μM) for 20 min. After washing, tissues were mounted in Vectashield (Vector). Fluorescence images were acquired using a Zeiss LSM510 Meta confocal laser scanning microscope (40× objectives). The primary antibody, anti-dFoxO antibody, was generated in rabbits using two peptides containing amino acids 2–16 (MDGYAQEWPRLTHTD) and amino acids 585–595 (AYPNSEPSSDS) (Eurogentec, Belgium) and diluted 1:500.

### Chromatin Immunoprecipitation

At 72 hr old, larvae were transferred and fasted overnight on 0.1× yeast medium. Then, lots of 30 larvae were collected and inverted in PBS to help subsequent fixation and homogenization. The ChIP protocol was performed as described by Teleman et al. (2008). Immunoprecipitations were performed using dFOXO antibody for the dFOXO ChIP and mock ChIP with preimmune serum. Experiments were each performed in three biological replicates. DNA enrichment was assessed by qRT-PCR analysis. dFOXO binding to the *4EBP* promoter was used as a positive control, and *sry* genomic regions were used as negative controls as in the work of Teleman et al. (2008). Primer sequences are available on request.

## Supplemental Data

Document S1. Three Figures and Supplemental Experimental Procedures
