# Supplementary material for: A Drosophila Insulin-like Peptide Promotes Growth during Nonfeeding States
Source: Dev Cell. 2009 Dec 15;17(6):874–84. doi: 10.1016/j.devcel.2009.10.009 (PMC2806523; doi:10.1016/j.devcel.2009.10.009)
Supplement: Document S1. Three Figures and Supplemental Experimental Procedures [file mmc1.pdf]

Developmental Cell *17*

Supplemental Data

A *Drosophila* Insulin-like Peptide Promotes Growth during Nonfeeding States

Maija Slaidina, Rénald Delanoue, Sebastian Gronke, Linda Partridge, and Pierre Léopold

Slaidina_Supplemental Material

Slaidina_Supplemental Figure S1

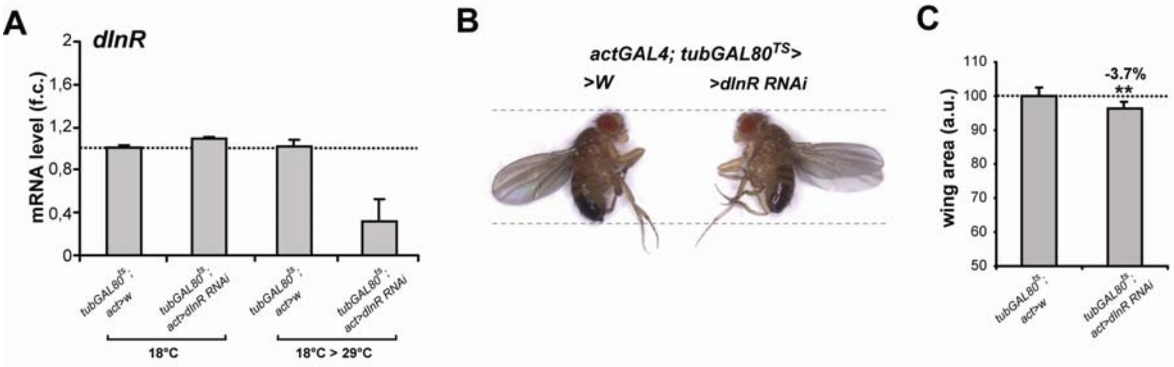

Supplemental figure S1: Organismal growth requires insulin signalling during pupal stages.
(A) Measurement of *dInR* expression by qRT-PCR from *tubGal80^{ts}; act>w* and *tubGal80^{ts}; act>dInR RNAi* pupae either kept at restrictive temperature (18°C) or transferred at 29°C at 120h AED to silence *dInR* expression (18°C>29°C). RNA extracts were made 12h after the temperature shift. Fold changes are relative to *tub-Gal80ts; act>w* animals under the same temperature shift program. Error bars represent SD.
(B) *tubGal80^{ts}; act>dInR RNAi* flies are smaller than *tubGal80^{ts}; act>w* when *dInR* is silenced during pupal stages by temperature shift-up experiment at 120h AED.
(C) Wing area of *tubGal80^{ts}; act>w* and *tubGal80^{ts}; act>dInR RNAi* under the same temperature shift programs.

Slaidina_Supplemental Figure S2

A 1st transcript

CGCGGATCCGAACACTGCGTTTGCTGGCTTTGATGAAA*CAGTGCACGTTTGCTTGTTGAGAGGAAAGGTT*
GTGTGCGGACGAATTTTTTTTTGAAAACATTAACCCTTACGTGGAATAAAAAAAA ***ATGAAATATTGCAAATTTT***
GCTGCAAAGCTGTGACTGGAGTAAAATTAATTCACGTGCCGAAGTGTGCTATTAAGAGAAAATTGTGGGAG
CAGAGCCTTGGGTGCAGCCTTGGTGAAAACTCCCAAATTTGTGATACCCACTTTAATGATTCGCAGTGGAA
GGCTGCACCTGCAAAAGGTCAGACATTTAAAAGGAGGCGACTCAACGCAGATGCCGTACCTAGTAAAGTG
ATAGAGCCTGAACCAGAAAAGATAAAAGAAGGCTATACCAGTGGGAGTACACAAACAGA **ATG**

2nd transcript

ATCAACTAACCATATACAAGTGCAGGTGGTAGTGATGGAAGAAACATAGATGGGTTTGATTCAAGTGGCAGCC
CCGACACAAGCGTCGGGTTCCGGCAAATCACCAATAGATGGCGTGCATAGGCGCGAAATTTGTATTAAATGC
CACTTTTGGTCGCTTGAAATGATACAAATAAATGAAACCATTATTATTATTTTAAAACGATTTCAACACGATCTTT
GAA**ATG**

B

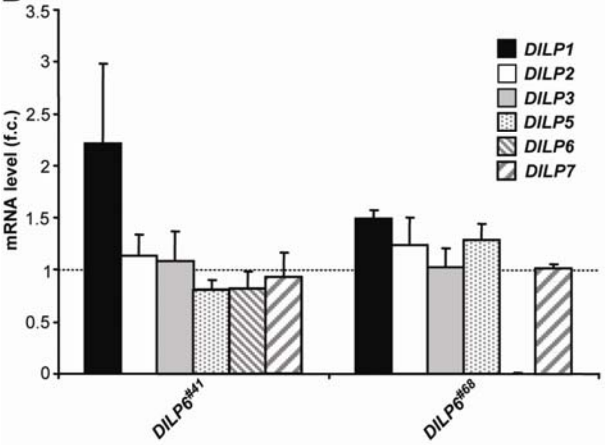

C

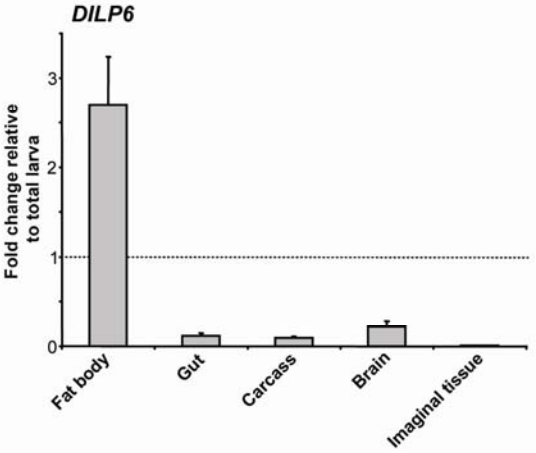

Supplemental figure S2: *DILP6* expression in the highest in the fat body.
(A) Sequence of the DILP6$^{\#41}$ locus. 5'RACE experiments reveal 2 transcripts. The first transcript is a fusion between part of KG004792 P element (underlined italic) and *DILP6* exon2 (arrow). This transcript includes the full DILP6 open reading frame (ORF) and an additional ORF encoding part of the transposase peptide (bold underlined italic). The second transcript shows a fusion between the adjacent genomic locus of the pole hole gene (*ph1*) (grey) and *DILP6* exon2, creating an additional upstream ORF (underline grey).
(B) Relative expression of *DILP* genes in *DILP6* mutants (*DILP6*$^{\#41}$ and *DILP6*$^{\#68}$) at wandering stage compared to control. Error bars represent SD.
(C) Relative expression of *DILP6* in fat body, gut, carcass, brain and imaginal tissues at wandering stage (110h AED). Fold changes are relatives to total larva RNA. Error bars represent SD

Slaidina_Supplemental Figure S3

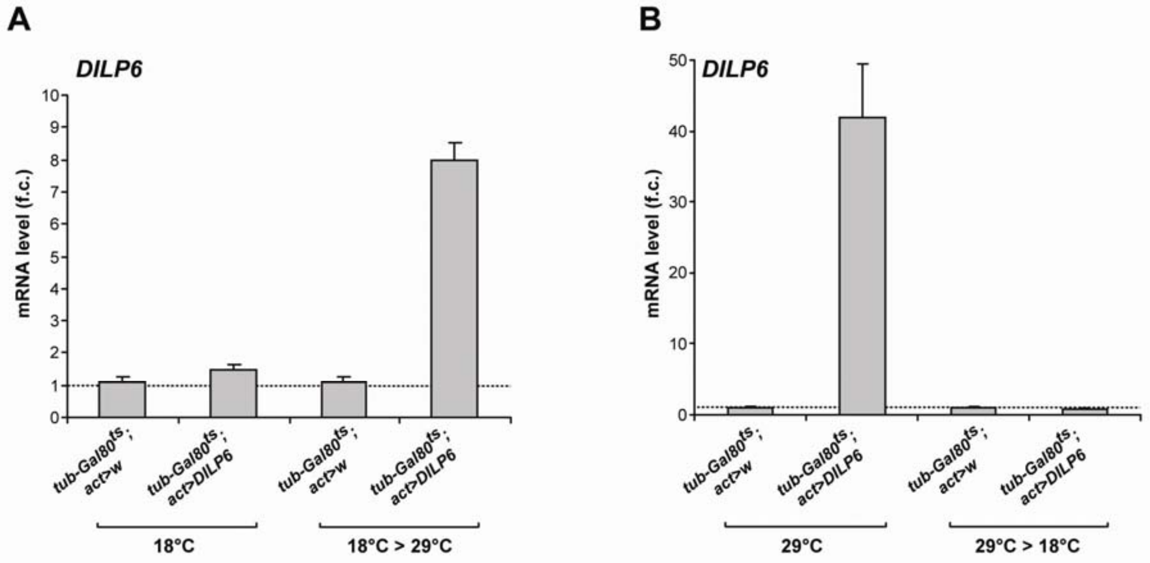

Supplemental figure S3: DILP6 overexpression is accurately induced or repressed by temperature shift-up and shift-down programs.
(A) Measurement of *DILP6* expression by qRT-PCR from *tubGal80^{ts}; act*>*w* and *tubGal80^{ts}; act*>*DILP6* pupae either kept at restrictive temperature (18°C) or put at 29°C at 120h AED to drive *DILP6* overexpression (18°C>29°C). RNA extracts were made 12h after the temperature shift. Fold changes are relative to *tubGal80^{ts}; act*>*w* animals under the same temperature shift program. Error bars represent SD.
(B) Measurement of *DILP6* expression by qRT-PCR from *tubGal80^{ts}; act*>*w* and *tubGal80^{ts}; act*>*DILP6* larvae either kept at non-restrictive temperature (29°C) or transferred at 18°C at 96h AED to block *DILP6* overexpression (29°C>18°C). RNA extracts were made 12h after the temperature shift-down. Fold changes are relative to *tubGal80^{ts}; act*>*w* animals under the same temperature shift program. Error bars represent SD.

SUPPLEMENTAL EXPERIMENTAL PROCEDURES

5´RACE

Total RNA was prepared from adult males of *DILP6*$^{\#41}$ mutants, *DILP6*$^{\#68}$ mutants and wild type controls. The 5´RACE was done using the FirstChoice® RLM-RACE Kit (Ambion) according to the instructions of the manufacturer. SOL8 was used in combination with the outer primer for the first round, SOL167 in combination with the inner primer in the second round of the nested PCR. PCR products were subcloned in pCRII-Topo vector (Invitrogen) and sequenced.

Nested PCR with primer combinations:
First round PCR:
a. SOL8/outer primer
b. SOL167/outer primer
Second Round PCR:
a. SOL167/inner primer
b. SOL165/inner primer

SOL8: GGGTGTGGCTGAGTGGTGG
SOL165: AGGGCCACCGTTCCGCTTAC
SOL167: CTTGCAGCACAAATCGGTTACG
